# A New Approach to Detect Congestive Heart Failure Using Short-Term Heart Rate Variability Measures

**DOI:** 10.1371/journal.pone.0093399

**Published:** 2014-04-18

**Authors:** Guanzheng Liu, Lei Wang, Qian Wang, GuangMin Zhou, Ying Wang, Qing Jiang

**Affiliations:** 1 School of Engineering, Sun Yat-sen University, Guangzhou, China; 2 Shenzhen Institutes of Advanced Technology, the Chinese Academy of Sciences, Shenzhen, China; University of Udine, Italy

## Abstract

Heart rate variability (HRV) analysis has quantified the functioning of the autonomic regulation of the heart and heart's ability to respond. However, majority of studies on HRV report several differences between patients with congestive heart failure (CHF) and healthy subjects, such as time-domain, frequency domain and nonlinear HRV measures. In the paper, we mainly presented a new approach to detect congestive heart failure (CHF) based on combination support vector machine (SVM) and three nonstandard heart rate variability (HRV) measures (e.g. *SUM_TD*, *SUM_FD* and *SUM_IE*). The CHF classification model was presented by using SVM classifier with the combination *SUM_TD* and *SUM_FD*. In the analysis performed, we found that the CHF classification algorithm could obtain the best performance with the CHF classification accuracy, sensitivity and specificity of 100%, 100%, 100%, respectively.

## Introduction

Autonomic dysfunction is a typical feature of chronic heart failure and is associated with severity of disease and prognosis in chronic heart failure (CHF) [1]. As a simple noninvasive technology, heart rate variability (HRV) analysis provides reliable information on autonomic modulation of heart rate, and it has been a valuable tool to understand psychopathological mechanisms of heart failure. Indeed, the significant difference of heart rate variability between patients with chronic heart failure and healthy people was widely reported in previous studies [2]. However, CHF is asymptomatic in its first stages. Therefore, early assessment of CHF severity is crucial to avoid the condition worsening and contribute to decrease medical cost.

The heart rate variability (HRV) measures have been mainly studied for the prognosis of the disease, in particular, as predictor of the risk of mortality. Several studies addressed the relationship between HRV and CHF [3]–[4]. For example, Nolan et al. (1998) prospectively showed that SDNN (standard deviation of all RR intervals) was a strong independent prognostic tool in CHF patients [5]. The low-frequency spectral component LF (ranging between 0.03 and 0.15 Hz) decreased in CHF patients with advanced disease and was related to the progression of the heart failure [6]. Guzzetti et al (2000) also suggested information content present in non-linear analysis of HRV in CHF patients has prognostic relevance independently from the time domain and spectral analysis of HRV [7]. However, HRV parameters were inevitably disturbed by its spontaneous fluctuation [8], respiration [9], motion artifacts [10], and ambulatory health-monitoring application and so on. As a result, some guide evidences that HRV analysis shouldn't form the primary basis for CHF assessment, because of its sensitivity and specificity.

Conventional, the New York Heart Association (NYHA) classification, which is a symptomatic functional scale, is one of the most widespread assessment methods of the severity of CHF [11]. As an objective evaluation, the comprehensive 2-D echocardiogram coupled with Doppler flow was also widely used for assessment of CHF [12]. However, an interesting question is whether HRV analysis may improve both sensitivity and specificity of ECG examination, thus providing a robust independent tool for CHF assessment.

Recently, a small number of attentions are being paid to CHF assessment based on heart rate variability (HRV) measurement. Asyali et al. (2003) adopted conventional time and frequency parameters of HRV and a Bayesian classifier to discriminate CHF disease with sensitivity and specificity rate of 81.82% and 98.08%, respectively [13]. Isler et al (2007) investigated the discrimination power of combining wavelet entropy and conventional HRV parameters by using the genetic algorithms and k-nearest neighbor classifier. The best performance was obtained with sensitivity and specificity rate of 100.00% and 94.74%, respectively [14]. Pecchia et al (2011) presented the CART algorithm and tree-like decision classifier to achieve CHF classification based on the conventional time-/frequency-parameters, including of two nonstandard feature, e.g. *ΔAVNN* (average of all RR intervals) and *ΔLF/HF* [15]. The result demonstrated the sensitivity and specificity rate were 89.7% and 100%, respectively. Although these studies reached interesting results, they all use superabundant HRV parameters, which could affect the discrimination sensitivity or increase the complex of classifier for the daily activity of clinicians.

In the paper, we presented a CHF patients' classification algorithm based on three new nonstandard HRV measures and support vector machine (SVM).

## Methods and Experiments

### Data

The dataset used in this work was obtained from an online and widely-used database, i.e. MIT/BIH database [16]. This study was approved by the Institutional Review Boards of Beth Israel Deaconess Medical Center (Boston, MA) and the Massachusetts Institute of Technology (Cambridge, MA). All subjects provided informed written consent and all data were de-identified. Patient records/information was anonymized and de-identified prior to analysis. Two RR interval database were chosen, including: 1) Thirty healthy subjects with a mean age of 49.33±19.77 years (range 40–72 years); 2) Seventeen patients with CHF in NYHA I–III with a mean age of 60.88±50.01 years (range 51–71 years). The data of healthy subjects was retrieved from the normal sinus rhythm RR interval database. The data for the CHF group was retrieved from the congestive heart failure RR interval database. The RR interval records are provided with beat annotations obtained by automated analysis with manual review and correction. According to the reference [9], the 5 minutes RR intervals were extracted from the beginning stage in the database. The original ECG records were digitalized at 128 samples per second.

### CHF Classification Algorithm

**Figure 1:**
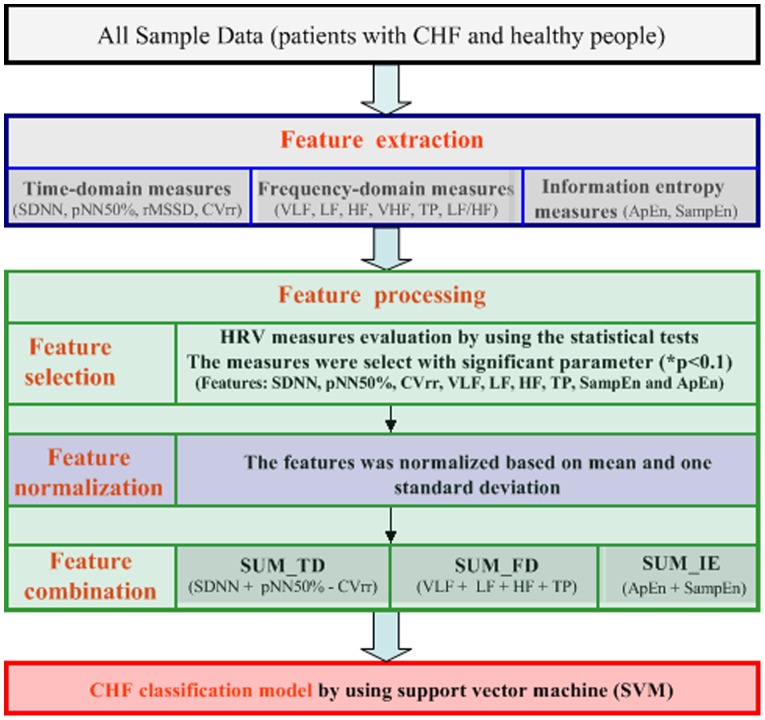
The CHF classification algorithm based on support vector machine.

The CHF classification model are comprised of short HRV feature computing, features processing, and Modeling based on support vector machine (SVM) as figure 1. Furthermore, the feature processing involves a three-part process of feature-selection, feature-normalization and feature-combination.

#### entropy-based measures of short term HRV

We extracted 5-min RR interval time series (RRITS) excerpts from the 24-h records and obtained 12 HRV features as the table 1.

**Table 1:** SELECTED HRV MEASURES.

HRV Measure	Description	Unit
**Four representative time domain measures were selected as following:**		
*SDNN*	One standard deviation of all normal sinus RR intervals over 5 minute	ms
*RMSSD*	Root mean square of the successive normal sinus RR interval difference over 5 minute	ms
*pNN50%*	percentage of successive normal sinus RR intervals longer than 50 ms during 5 minute	%
*CVrr*	The coefficient variation of all normal sinus RR interval	/
**The six frequency domain measures were computing based on nonparametric power spectral density analysis and fast fourier transform (FFT) ** [**17**] **, as following:**		
*VLF*	Total spectral power of all normal sinus RR intervals 0–0.04 Hz	ms^2^
*LF*	Total spectral power of all normal sinus RR intervals 0.04–0.15 Hz	ms^2^
*HF*	Total spectral power of all normal sinus RR intervals 0.15–0.4 Hz	ms^2^
*VHF*	Total spectral power of all normal sinus RR intervals 0.4–1.0 Hz	ms^2^
*TP*	Total spectral power of all normal sinus RR intervals 0–0.4 Hz	ms^2^
*LF/HF*	The ratio of LF to HF	
**Information entropy is becoming a usual tool for characterizing the RR interval series, two measures were obtaining as following:**		
ApEn	Approximate entropy as the following equation (4)	/
SampEn	Sample entropy as the following equation (7)	/

The ‘/’ means dimensionless unit.

The *ApEn* and *SampEn* were computed based on the following equation (1–5).

First It is assumed that RR time series is the data set {*RR* (*k*), *k* = 1, 2…*N*} with length N. then, the phase space is reconstructed by choosing two parameters: the embedding dimension (*m*) and the delay (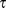) [18]. The delay (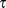) is 1 beat and embedding dimension (*m*) is 2 in the paper. The (*N-m+1*) templates are composed as follows:
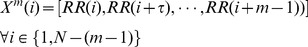
(1)


The recurrence matrix and distance between matrices are calculated as equation (2) and (3):
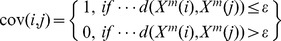
(2)

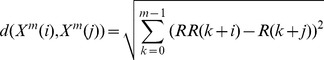
(3)


Where 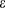 is constant, (e.g. one standard deviation from RR time series).

Thus, the Approximate Entropy is defined as:
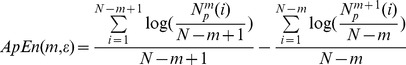
(4)


Then, for each 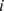:
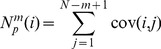
(5)


Since approximate entropy indicates more similarity than it's truly one for finite time series. To reduce the bias caused by it matching, sample entropy (*SampEn*) was developed to quantify heart rate variability [19]. The equations (2) and (4) are adjusted:
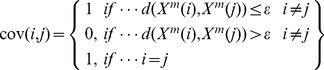
(6)


Then the SampEn is computed as following equation:
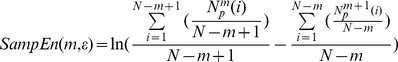
(7)


#### Three short-term nonstandard HRV measures

Feature processing in a high-dimensional data space is often used to decrease the computational cost and improve the accuracy during the classification process [20]. The feature processing procedure is included of feature-selection, feature-normalization and feature- combination in our study as following:

Step-1: In contrast to the feature-extraction techniques, feature selection is used to identify the variables that do not contribute to the classification process. In the paper, we assessed all the front 12 HRV measures (also known as features) by using the statistical method (with SPSS software, vision 14 (SPSS Inc, Chicago, IL, USA)). First, mean and standard deviations is used to evaluate the mean absolute error between the healthy group and the CHF group. Then, two datasets are compared by using two sample t-tests for each HRV feature. The significance level chosen was α = 0.1. The bigger the significance parameter (*p) is, the smaller the contribution is. At last, if the significance parameter (*p) is greater than 0.1, the HRV feature was rejected. Table 2 demonstrated that three HRV features (e.g. *rMSSD*, *VHF* and *LF/HF*) were deleted with the significance parameter (*p>0.1).

**Table 2:** HEART RATE VARIABILITY (HRV) MEASURES BETWEEN CHF DISEASES AND HEALTHY PEOPLE.

HRV features	CHF group (mean± SD)	Health group (mean ± SD)	significance parameter (*p value)
*SDNN*	298.2±124.1	81.3±49.7	0.0001
*pNN50%*	13.1±5.45	2.90±1.65	0.0001
*rMSSD*	380.4±268.9	480.0±242.0	0.339
*CVrr*	3.79±3.60	8.59±9.44	0.01
*VLF*	5.72±2.08	7.30±2.61	0.073
*LF*	2.59±0.96	3.51±1.66	0.062
*HF*	0.64±0.26	0.86±0.42	0.056
*VHF*	0.38±0.14	0.43±0.14	0.144
*TP*	9.33±3.37	12.1±4.46	0.058
*LF/HF*	4.09±0.35	4.07±0.31	0.175
*ApEn*	0.36±0.28	1.16±0.38	0.0001
*SampEn*	0.33±0.28	1.21±0.40	0.0001

(Shaded if significant difference with the reference value at *p>0. 1).

Step-2: Feature normalization was used to independently normalize each feature component to the [0 1] range [21]. Feature normalization could reduce the differences among HRV measures. It assumes that the feature vector X {*X* (*i*), *i* = 1, 2…N}. The normalization's feature (Y) was obtained as following equation:
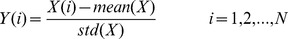
(8)

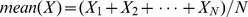
(9)

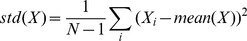
(10)


Step-3: our study supposed that the same type of feature (e.g. time domain, frequency domain and information entropy) has extremely similar difference between CHF group and healthy group. Thus, feature-combination could further reduce the dimensionality and computational cost. Three new nonstandard features are combined of the nine features (e.g. *SDNN*, *pNN50%*, *CVrr*, *VLF*, *LF*, *HF*, *TP*, *ApEn* and *SampEn*) as the following equation:

The nonstandard time-domain feature was computing as following:
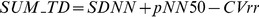
(11)


The nonstandard frequency-domain feature was computing as following:
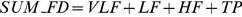
(12)


The nonstandard non-line feature was computing as following:
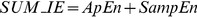
(13)


#### CHF classification algorithm based on support vector machine (SVM) and three nonstandard features

As one of the most popular classifiers, Support vector machine (SVM) aims to find a hyperplane that can separate two classes of data with maximal margin [22]. The *n*-dimensional feature vector is *x* and the bias *b* belongs to {−1, 1}. The decision function *f(x)* was computed as following:
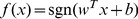
(14)


The weight vector *w* and bias *b* representing a separating hyperplane. The SVM finds an optimal separating margin by solving the following optimization task:
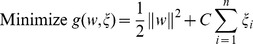



Subject to:
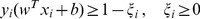
(15)


Where, the parameters 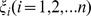 were the slack variables, the constant parameter *C* is obtained by a cross-validation method. The polynomial kernel function was chosen as following equation:
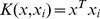
(16)


### K nearest-neighbor (KNN) classifier for CHF classification

To evaluate our algorithm (section II_B) performance, a k-nearest neighbor classifier [14] was also investigated in the paper. By comparison among previous CHF classification methods, we found the K nearest-neighbor (KNN) classifier [14] has the best performance. Moreover, the different inputs form time domain, frequency domain and non-line features were considered to obtain optimal CHF classification performance. The KNN classifier with the combination of different features was also studied in terms of SEN and AUC.

### Performance measurements

To measure the performance of CHF classification algorithm, we use the confusion matrices [23]. From these matrices, we compute the widely used measures reported in table 3 for binary classification in order to enable the comparison of our method with others. The software program Mathworks Matlab version 7 (R2007b) (The MathWorks, Natick, MA, USA) is used for data processing in the paper. We did the statistical tests (with SPSS software, vision 14 (SPSS Inc, Chicago, IL, USA)) for HRV measures choice. Where two datasets are compared, we perform two sample t-tests for each individual method. The significance level is chosen as α = 0.1.

**Table 3:** CHF CLASSIFICATION PERFORMANCE MEASURES.

Measure (Abbreviation)	Formula
Accuracy (*ACC*)	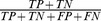
Precision (*PRE*)	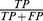
Sensitivity (*SEN*)	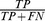
Specificity (*SPE*)	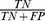
Area Under the Curve (*AUC*)	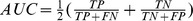

TP: Number of CHF patients detected and TN: number of normal subject detected. FP: Number of normal subject incorrectly labeled as CHF and FN: number of CHF patients incorrectly labeled as normal.

## Results

### The performance of three nonstandard features

**Figure 2:**
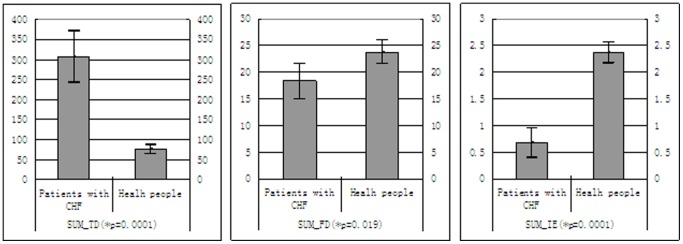
Three nonstandard features between patients with CHF and healthy people; Mean ±one standard deviation was plotted. SUM_TD was a nonstandard time domain feature; SUM_FD was a nonstandard frequency domain feature; SUM_IE was a nonstandard non-line feature.

The fig. 2 demonstrates the difference of three nonstandard features (*SUM_TD*, *SUM_FD* and *SUM_IE*) between patients with CHF and healthy people. In patients with CHF, the *SUM_TD* (307.47±129.0 versus 75.60±44.86), is significant higher than one of healthy people with the significant level (*p<0.0001). By comparison with all time-domain features, the combinational feature (*SUM_TD*) has bigger difference. In patients with CHF, the *SUM_FD* (18.29±6.60 versus 23.78±8.79), the *SUM_IE* (0.68±0.56 versus 2.37±0.77) are significant lower than those of healthy people with the significant parameter (*p) of 0.019, 0.0001, respectively. In comparison with all frequency, the *SUM_FD* has more small significant parameter (*p). The *SUM_IE* has also bigger difference than single information entropy feature (e.g. *ApEn* and *SampEn*).

Thus, three nonstandard features significantly magnify the difference between patients with CHF and healthy people. For example, the frequency feature (*SUM_FD*) has more significantly diffidence (*p<0.019) that all frequency features (e.g. *VLF*, *LF*, *HF*, *LF/HF*, *TP* and *VHF*). Thus, the feature-processing obviously magnifies the difference, and reduces the dimension and the computational cost of the CHF disease classification model based on SVM classifier.

### CHF classification performances among different feature combination

The study found that single nonstandard feature was still unable to separate patients with CHF from healthy people with 100% accuracy. In comparison, the *SUM_IE* is best classification parameter with the best accuracy of 95.74%. The *SUM_FD* has worst classification accuracy of 59.57%.

**Figure 3:**
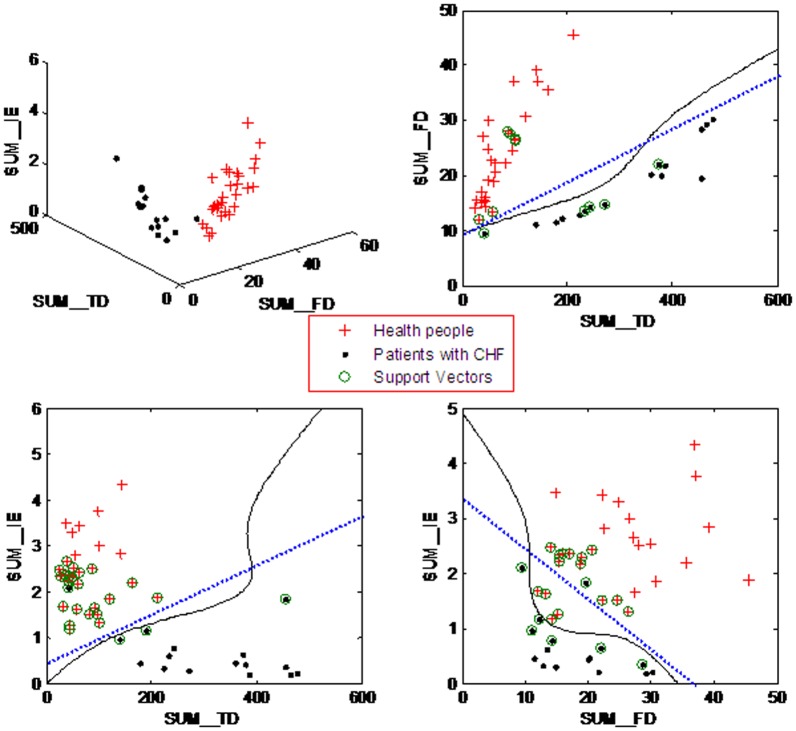
The SVM classifier from different input feature vectors. SUM_TD was a nonstandard time domain feature; SUM_FD was a nonstandard frequency domain feature; SUM_IE was a nonstandard non-line feature.

The figure 3 demonstrates four kinds of CHF classification model based different input feature vectors. The blue-dotted lines (fig. 3) show the linear separating curves for three kinds of input feature vectors (e.g. *SUM_TD*, *SUM_FD*; *SUM_TD*, *SUM_IE*; and *SUM_IE*, *SUM_F*D). The results suggest that the input feature vector with *SUM_TD* and *SUM_FD* was linear completely separable. Obviously, if the three nonstandard features are all chosen as input feature vector, the CHF classification model should also be linear separable. The input feature vector with *SUM_TD* and *SUM_IE* aren't linear separable with two linear inseparable samples. The input feature vector with *SUM_FD* and *SUM_TD* is also linear inseparable with five linear inseparable samples. Moreover, three black curves in the fig. 3 are the separating curves of SVM based on polynomial kernel. And the result implies the SVM classifier' effect of the polynomial kernel is better than one of linear kernel.

### CHF classification algorithm

By comparison with K nearest-neighbor (KNN) classifier [14] (as table 4), our algorithm could obtain better performance of CHF classification. The results demonstrated that the KNN classifier with all 12 features has the lowest discrimination power in all terms (*ACC*, *PER*, *SEN*, *SPE* and *AUC*). The performance of different CHF classification models based is demonstrated as table 5. The result suggests that the optional classification models could achieve accuracy (*ACC*), precision (*PRE*), sensitivity (*SEN*), specificity (*SPE*), area under the curve (*AUC*) of 100.00%, 100.00%,100.00%, 100%, 100%, 100.00% as following input feature vector: (a) *SUM_TD*, *SUM_FD* and *SUM_IE*; (b) *SUM_TD* and *SUM_FD*; (c) all 12 features. However, considered to the computation cost, the CHF classification model with the input features (*SUM_TD* and *SUM_FD*) is chosen in the study. Besides, other input feature vectors (e.g. *SUM_TD* and *SUM_IE*; *SUM_IE* and *SUM_FD*) also obtain preferably performance with the classification accuracy of 97.98%.

**Table 4:** THE K-NEAREST-NEIGHBOR CLASSIFER FOR CHF CLASSIFICATION.

Feature combination	*ACC* (%)	*PRE* (%)	*SEN* (%)	*SPE* (%)	*AUC* (%)
All features	57.45	70.59	44.44	75	59.72
Time domain features	89.36	76.47	92.86	87.88	90.37
Frequency domain features	59.57	70.59	46.15	76.19	61.17
Non-line features	80.85	88.24	68.18	92	80.09
Time domain and non-line features	91.49	94.12	84.21	96.43	90.32

Time-domain features were *SDNN*, *rMSSD*, *pNN50%* and *CVrr*; Frequency domain features were *VLF*, *LF*, *HF*, *VHF*, *TP* and *LF/HF*; The non-line features were *ApEn* and *SampE*;

**Table 5:** THE PERFORMANCE OF CHF classification MODEL BASED ON SVM.

Feature combination	*ACC* (%)	*PRE* (%)	*SEN* (%)	*SPE* (%)	*AUC* (%)
***SUM_TD SUM_FD SUM_IE***	100.00	100	100.00	100	100.00
***SUM_TD SUM_FD***	100.00	100	100.00	100	100.00
***SUM_TD*** **, ** ***SUM_IE***	97.98	100	94.12	100	97.06
***SUM_IE*** **, ** ***SUM_FD***	97.98	100	94.12	100	97.06
***SUM_TD***	91.49	88.24	88.24	93.33	90.78
***SUM_FD***	59.57	47.06	44.44	68.97	56.70
***SUM_IE***	95.74	88.24	100.00	93.75	96.88
All 12 features*	100.00	100	100.00	100	100.00

All 12 features were *SDNN*, *rMSSD*, *pNN50%*, *CVrr*, *VLF*, *LF*, *HF*, *VHF*, *TP*, *LF/HF*, *ApEn* and *SampE*. *SUM_TD* was a nonstandard time domain feature; *SUM_FD* was a nonstandard frequency domain feature; *SUM_IE* was a nonstandard non-line feature.

The support vector is computed by SVM classifier:
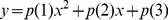
(17)


Where the parameters (*p*) is: [0.0001 0.0208 9.3036]; the parameter (*x*) is *SUM_TD*; the parameter (*y*) means output value from the model.

Thus, the CHF classification model is obtained by SVM:
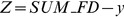
(18)


If *Z*<0 represents CHF patients.

## Discussion and Conclusion

In this study, we aim to investigate the CHF classification power from three nonstandard short-term HRV features (e.g. *SUM_TD*, *SUM_FD* and *SUM_IE*) based on SVM classifier. The CHF classification model (equation 18) we presented could achieve to discriminate CHF patients from healthy people with the accuracy values of 100%. Moreover, the three nonstandard HRV features enhance the difference between healthy people and patients with CHF, and contribute to CHF classification.

Bilchick and Berger reported that a depressed *SDNN* (<70 ms and <30 ms, respectively, for long-term and 5-min records) is significantly associated with increased mortality [24]. In our study, the depressed *SUM_IE* (<1.18) is significantly associated with CHF diseases with the significant parameter (*p<0.001). The *SUM_FD* had worst CHF classification performance with the accuracy value of 59.57%. However, when the input features chose *SUM_TD* and *SUM_IE*, the classification accuracy achieved to 100%. The high complementarity between nonstandard frequency-domain feature (*SUM_TD*) and nonstandard time-domain feature (*SUM_IE*) have contributed to improve the CHF classification's performance.

Compared to the other studies in table 6, we found that the long-term HRV measures haven't contributed to improve the CHF classification's performance. The KNN classifier from the paper has a bit worse performance than the KNN classifier form Isler et al. The possible reason is that the study form Isler et al chose more HRV measures (e.g. Wavelet entropy and Poincare plot). The study form Isler et al has more sample numbers with the 29 patients and 54 healthy subjects. Obviously, the SVM classifier from the paper has best performance for CHF classification.

**Table 6:** CHF CLASSIFICATION PERFORMANCE ASSESSMENT OF DIFFERENT CLASSIFIERS.

	ACC	PRE	SEN	HRV measures	Time
Bayesian from Asyali [13]	93	95	82	*SHRV*	**Long-term**
RT from Pecchia [15]	96.4	100	89.7	*ΔLF/HF*, *SHRV* and *ΔAVNN*	**Long-term**
KNN from Isler [14]	96.4	91	100	*WE*, *PP* and *SHRV*	**Short- term**
KNN from the paper	91.49	94.12	84.21	Time domain and non-line features	**Short- term**
SVM from the paper	100	100	100	*SUM_TD*, *SUM_FD*	**Short- term**

KNN: A *k*-nearest-neighbor; RT: regression tree; WE: Wavelet entropy, PP: Poincare plot; *SHRV*: standard HRV measure, including conventional time and frequency measures such *SDNN*, *rMSSD*, *pNN50%*, *VLF*, *LF*, *HF*, *VHF*, *TP*, *LF/HF*, etc; *AVNN*: average of all RR intervals. Time-domain and non-line features were *SDNN*, *rMSSD*, *pNN50%*, *CVrr*, *ApEn* and *SampEn*; *SUM_TD* was non-standard time domain feature; *SUM_FD* was non-standard frequency domain feature.

In the study, we could draw some conclusions:

1. Three short-term nonstandard HRV measures (e.g. *SUM_TD*, *SUM_FD* and *SUM_IE*) we presented are suited to CHF classification. In fact, the nonstandard HRV features are better than conventional standard HRV measures in CHF classification.
2. The SVM classifier with the combination of nonstandard time-domain and nonstandard frequency-domain have the highest discrimination power with the classification accuracy, precision, sensitivity, specificity of 100%, 100%, 100%, 100%, respectively. In fact, the SVM classifier is superior to KNN classifier. The most serious problem for KNN classifier is the feature chooses and combination.
3. The study also found that the short-term HRV measures could substitute for long-term HRV measures in the CHF classification. The CHF classification performance with long-term HRV measures is not significantly above one with short-term HRV measures.

Finally, the proposed CHF classification algorithm meets all our requirements because it is fully noninvasive, low-cost and high accuracy method that provides an objective classification for classification of CHF.
